# Development of an Analytical Method for the Rapid Quantitation of Peptides Used in Microbicide Formulations

**DOI:** 10.1007/s10337-014-2777-7

**Published:** 2014-10-17

**Authors:** Yufei Chen, Sidi Yang, Emmanuel A. Ho

**Affiliations:** Laboratory for Drug Delivery and Biomaterials, Faculty of Pharmacy, University of Manitoba, Apotex Centre, 750 McDermot Ave, Room 329, Winnipeg, MB R3E 0T5 Canada

**Keywords:** Intravaginal gel, Protein degradant, Reversed-phase HPLC, Tris-tricine SDS-PAGE, Separation

## Abstract

Recently, a growing number of macromolecules such as peptides and proteins have been formulated into various microbicide formulations for the prevention of sexually transmitted infections. However, a fast and reliable high-throughput method for quantitating peptide/protein in polymer-based microbicide formulations is still lacking. As a result, we developed and validated a reversed-phase high-performance liquid chromatography method for the quantitation of gp120 fragment and LL-37 simultaneously in various microbicide gel formulations. This method was capable of detecting a limit of linearity (regression coefficient of 0.999) for gp120 fragment and LL-37 within a range of 0.625–80 and 1.25–80 µg mL^−1^, respectively. The lower limit of quantification for gp120 fragment and LL-37 was 1.14 and 0.31 µg mL^−1^, respectively. Method validation demonstrated acceptable intra- and inter-day RSD % (<5 %) and accuracy (95.67–100.5 %). Formulating both peptides into polymeric pharmaceutical gel formulations showed high extraction efficiency (in the range of 95.90 ± 3.03 to 111.45 ± 2.51 %). Using this method, we were able to separate and identify the forced degraded products from both peptides simultaneously without affecting the quantitation of both peptides in the polymeric dosage forms. Furthermore, this method was able to detect and separate degradants that were unable to be revealed using gel eletrophoresis.

## Introduction

Sexually transmitted diseases (STDs) including human immunodeficiency virus (HIV)/acquired immunodeficiency syndrome (AIDS) present a huge threat globally, especially to women [1]. Women are biologically two times more likely than men to be infected by HIV via heterosexual intercourse. For example, after sexual intercourse, semen remains within the female genital tract for prolonged periods of time, increasing exposure to HIV. In the absence of an effective vaccine, microbicides are being developed for vaginal and rectal application as a strategy to prevent and/or reduce sexual transmission of HIV. The emergence of microbicides has greatly improved the feasibility for women to protect themselves because the use of microbicides is discreet without requiring co-operation or consent by their sexual partners [2, 3]. Microbicides have been formulated into various dosage forms including gels, films, tablets, and vaginal rings [4–6]. The active microbicidal agent can vary from small molecule drugs [7] to large biomacromolecules such as peptides and proteins [8]. Peptides, as a class of anti-microbicidal agents, have gained extensive attention recently due to their highlighted properties such as their broad anti-microbial and immunomodulatory effects in comparison to chemical agents [9, 10].

However, the lack of reliable and rapid quantification methods for this class of microbicides presents as a great inconvenience to their researchers, especially analysts. Although there are several existing methods for protein/peptide quantification such as spectrophotometry, western blot, and enzyme-linked immunosorbent assay (ELISA), their individual limitations hinder them from satisfying the needs of practical applications. For example, the commercially available BCA/Bradford assay based on spectrophotometry lacks specificity and sensitivity. These assays cannot distinguish between different proteins/peptides, and the lower limit of quantification (LLOQ) for the BCA assay that can be achieved without interference is approximately 1.23 µg mL^−1^ [11]. Furthermore, the accuracy of these assays are significantly reduced when approaching the LLOQ and the results can be inconsistent due to interference by impurities or the presence of other substances, e.g., excess salts, surfactants, polymers, etc. [11].Western blot has good specificity in identifying the target protein/peptide. It is a semi-quantitative method and is time-consuming, especially for the detection of small peptides due to the much higher degree of cross-linked gel matrix required, thus resulting in significantly longer electrophoresis run-time. ELISA is a reliable method with both specificity and accuracy, but the sample analysis is generally time-consuming requiring optimization of appropriate antibody/antigen incubation and can be expensive due to the need of antibodies with high specificity, thereby affecting its efficiency for analyzing large quantities of pharmaceutical samples. In contrast, reversed-phase high-performance liquid chromatography (RP-HPLC) has good precision (<2 %), acceptable selectivity and sensitivity (as low as 0.33 μg mL^−1^), relatively inexpensive (e.g., does not require the use of antibodies) and is less time-consuming than traditional protein analysis methods (e.g., gel electrophoresis) [11, 12].

In the current study, our group has developed a rapid and sensitive RP-HPLC quantification method for two peptides, gp120 fragment (encompassing the CD4 binding site) and LL-37, with potential applications in microbicide development. To the best of our knowledge, this is the first paper focusing on developing an accurate, rapid, robust and convenient analysis method that can be used to analyze these microbicidal peptides in polymeric gel formulations. LL-37 is the only cathelicidin host defense peptide found in humans [13, 14]. LL-37 has been shown to exhibit antibacterial [15], anti-fungal [16], anti-viral [17, 18], wound healing [19, 20], anticancer [21, 22], and immunomodulatory activity [23]. Glycoprotein 120 (gp120) is part of the HIV envelope protein and its fragment (target analyte in current study) has been used for the induction of anti-HIV neutralization antibodies [24, 25]. Although there are published literatures reporting the use of RP-HPLC for the purification of LL-37 [26, 27], to the best of our knowledge, there is no validated RP-HPLC method for determining the concentration of LL-37 or gp120 fragment alone or simultaneously. The current study is the first to apply a validated RP-HPLC method for the simultaneous quantification of LL-37 and gp120 fragment in polymeric gel dosage forms. Since peptides are generally unstable under certain environmental conditions resulting in degradation and/or aggregation, we further evaluated the application of the current RP-HPLC method for identifying the degradation or aggregation products of both peptides. This method is rapid, robust and convenient when developing novel microbicide gel formulations.

## Materials and Methods

### Materials

Water and acetonitrile (HPLC grade) were purchased from EMD (Mississauga, ON, Canada). LL-37 (4,493.33 Da, LLGDFFRKSKEKIGKEFKRIVQRIKDFLRNLVPRTES) and gp120 fragment (2,138.56 Da, KQFINMWQEVGKAMYAPP) were custom synthesized by CPC Scientific Inc. (Sunnyvale, CA, USA) and Biomatik Corp. (Cambridge, ON, Canada), respectively. Hydroxyethylcellulose (HEC) (Natrosol™, 250 HX PHARM) was a kind gift from Ashland Inc. (Covington, KY, USA). Hydroxypropyl methylcellulose (HPMC, Methocel E4 M Premium) was kindly supplied by The Dow Chemical Company (Calgary, Alberta, Canada) and hydroxypropyl cellulose (HPC) (4,000–6,500 cps) was purchased from Spectrum Chemical Manufacturing Corp. (New Brunswick, NJ, USA). Glycerol, trifluoroacetic acid (TFA), acetic acid and dipotassium phosphate were purchased from Fisher Scientific (Ottawa, ON, Canada).

### RP-HPLC Method

Stock solutions of both peptides (1 mg mL^−1^) were prepared in water. Working standard solutions were prepared from the stock solutions covering the range of 0.625–80 µg mL^−1^ (*n* = 6). Quantification of gp120 fragment and LL-37 was performed using a gradient RP-HPLC method. Analysis was performed using a XBridge™ BEH300 C18 column (300 Å, 5 µm 4.6 × 150 mm; Waters) with a Symmetry C18 guard column (300 Å, 5 µm, 3.9 × 20 mm; Waters), fitted to a Waters^®^ Alliance^®^ HPLC system equipped with Waters^®^ 2690 Separations module and Waters^®^ 996 Photodiode Array detector. Mobile phase A consisted of 0.1 % TFA in 10 % acetonitrile in HPLC grade water. Mobile phase B consisted of 0.85 % TFA in 90 % acetonitrile in water. A gradient A/B was applied to elute and analyze the peptides: A/B from 85:15 to 20:80 in 6 min, followed by maintaining at 20:80 for 2 min and re-equilibration of the column at 85:15. Total run was 10 min for each 200 μL injection. Flow rate was 2.0 mL min^−1^, UV detection was set at 210 nm, and column temperature was maintained at 60 °C. The retention time for gp120 and LL-37 were approximately 2.7 min and 4.1 min, respectively. The linear calibration curves for gp120 and LL-37 were obtained in the range of 0.625–80 µg mL^−1^ (*R* ^2^ > 0.999) and 1.25–80 µg mL^−1^ (*R* ^2^ > 0.999), respectively (*n* = 6).

### Preparation and Extraction of LL-37/gp120 from Polymeric Microbicide Gels

Approximately 1 g of HEC, HPMC, or HPC (2 %) was added to 40 mL of KH_2_PO_4_ (pH 7.4) and stirred at 4 °C for 2 h until dissolved. To adjust the viscosity of the gel to be similar to gels used for microbicide evaluation (e.g., ~2,736 cP) [28], 5 g of glycerol (10 %) was added and stirred until it was uniformly dispersed. Water was used to dissolve gp120 and 20 % acetic acid was used to dissolve LL-37 to make a concentration of 1 mg mL^−1^. Each peptide was mixed with the gels at a weight ratio of 1:1:4,000 (gp120:LL-37:gel) and stirred at 4 °C for 2 h resulting in a preparation containing 1 % HEC, HPMC, or HPC gel loaded with gp120 (0.025 %, w/w) and LL-37 (0.025 %, w/w).

For the extraction of peptides from polymeric gels, approximately 200 mg of the gel was weighed into a 5-mL volumetric flask and filled to volume with HPLC grade water. The sample was vortexed at room temperature for 30 s (three runs of 10 s each) and sonicated on ice for another 20 s (two runs of 10 s each). The extraction samples were then analyzed on RP-HPLC. To determine the extraction efficiency (assayed peptide concentration in the gel divided by the theoretical concentration in the gel), blank gels were spiked with known amounts of gp120 and LL-37 followed by the extraction procedure as described above.

### Forced Heat-Stress Studies

Both gp120 and LL-37 were exposed to various environmental conditions to force degrade the peptides. Since these peptides may be used for microbicide development, vaginal fluid simulant (VFS; 60 mM NaCl, 25 mM KOH, 3 mM Ca(OH)_2_, 6.6 mM urea, 28 mM glucose, 22 mM lactic acid, 16.7 mM acetic acid, 1.7 mM glycerol, pH 4.2) [29] was used for the stability studies. Both peptides were freshly dissolved in VFS separately to yield a 1 mg mL^−1^ solution. Aliquots of both peptides (200 µL) were then heat-stressed using a heating block at 95 °C for up to 8 h. The samples were then allowed to cool down to room temperature and stored at −20 °C prior to analysis. Non-heat-stressed samples were immediately stored at −20 °C after dissolving the peptides. The collected samples were analyzed either using the same RP-HPLC method described above or using tris-tricine SDS-PAGE [30]. For tris-tricine SDS-PAGE, stressed and non-stressed peptides (10 µg) were mixed with 5 µL of 3× sample loading buffer without 2-mercaptoethanol and loaded on to a tris-tricine gel, containing a stacking gel (4 % *T*, 3 % *C*, which *T* is for total acrylamide concentration and *C* is for crosslinker concentration), spacer gel (10 % *T*, 6 % *C*), and a separating gel (16 % *T*, 6 % *C*). Electrophoresis was performed at room temperature with an initial voltage of 30 V until the samples completely migrated into the spacer gel, followed by four incremental increases in voltage (15 V per increment) to a final voltage of 90 V until the tracking dye reached the bottom of the gel. After 5 h of electrophoresis, resolved peptides and their degradation/aggregation products were visualized using a rapid Coomassie Blue R-250 staining method [31]. Target degradation/aggregation peptide bands were then carefully cut from the gels and recovered by electro-elution. Briefly, gel slices containing the aggregation bands of interest (combined from 6 to 8 gels to increase recovery) were encased in a Spectra/Por^®^ Micro Float-A-Lyzer dialysis device (0.5–1 kDa MWCO Spectrum Laboratories, Inc., Compton, CA, USA) and immersed in transfer buffer (25 mM tris, 192 mM glycine, 20 % methanol v/v, pH 8.3). Electro-elution was conducted at 100 V for 1.5 h to ensure complete migration of peptide samples out of the gel matrix. After overnight dialysis against PBS (pH 7.4) at 4 °C to remove residual SDS, samples were freeze-dried using a benchtop freeze dry system (Labconco^®^ FreeZone 2.5 Liter). Lyophilized peptides and their aggregation products were re-solubilized in 250 µL double distilled water and immediately subjected to RP-HPLC analysis. Heat-stressed peptides were then used to spike HPC, HPMC, and HEC gels, and peptide extraction/RP-HPLC analysis was performed as described earlier.

### Statistical Analysis

Data are expressed as the mean ± standard deviation (SD) from the obtained results. Student’s *t* test was performed using GraphPad Prism version 6.0c (GraphPad Software, La Jolla California USA). *P* < 0.05 was considered as statistically significant.

## Results

### Method Validation

Linearity was determined from a calibration curve using standards of gp120 and LL-37 solutions in the range of 0.625–80 μg mL^−1^ in water and with six replicates. Linear relationship was observed between the peak area and the concentrations of peptides in water as reported in Table 1 with a regression coefficient of 0.999. The lower limit of detection (LLOD) and LLOQ were obtained as defined by the International Conference on Harmonisation (ICH) Topic Q2B [32] using the slope (*b*) of the calibration curve and the standard deviation, *Sa*, of the intercept (*a*) using the equations below:

**Table 1:** Method validation of RP-HPLC for gp120 fragment and LL-37

Conc. (μg mL^−1^ )	*R*	Equation	Mean ± SD		% RSD		Accuracy (%)		LLOD (μg mL^−1^ )	LLOQ (μg mL^−1^ )
			Intra-day	Inter-day	Intra-day	Inter-day	Intra-day	Inter-day		
gp120										
5	0.999	*Y* = 97,794.50 (± 1,128.59) × *X* + 8,627.98 (± 3,050.39)	4.84 ± 0.12	5.02 ± 0.10	1.27	0.47	96.38	100.4	0.10	0.31
15			14.97 ± 0.32	15.04 ± 0.33	0.28	0.23	100.36	100.5		
40			40.02 ± 0.17	39.93 ± 0.47	0.37	0.43	99.87	99.87		
LL-37										
5	0.999	*Y* = 64,831.25 (± 682.50) × *X* + 14,115.90 (± 7,384.79)	4.91 ± 0.42	5.02 ± 0.31	1.45	0.87	95.67	98.97	0.38	1.14
10			15.05 ± 0.37	15.01 ± 0.28	0.93	0.88	97.45	98.45		
40			39.93 ± 0.26	40.04 ± 0.32	0.82	0.21	97.23	100.23		

*R* regression coefficient, *LLOD* lower limit of detection, *LLOQ* lower limit of quantification

$$ {\text{LLOD}}=3.3 \times \frac{Sa}{b} $$

$$ {\text{LLOQ}}=10 \times \frac{Sa}{b} $$

Accuracy values were determined via the following equation and were always within the acceptable limits (±5 %) at all validation concentrations (Table 1).

$$ {\text{Accuracy}}\,\,\left({\%}\right)=\frac{{({\text{True value}}-{\text{Measured value}})}}{\text{True value}} \times 100 $$

Precision was determined from three standard calibration curves. Two were from the same day for intra-day precision and the third one was from a different day for inter-day precision. Intra- and inter-day precision was evaluated by analyzing both peptides at low (5 μL mL^−1^), medium (15 μL mL^−1^), and high (40 μL mL^−1^) concentrations (*n* = 6, Table 1). The relative standard deviation (%RSD) values were calculated for each concentration. The precision was found to be acceptable (<5 %), with the %RSD values of intra-day precision ranging 0.28–1.27 % for gp120 and 0.85–1.45 % for LL-37 as well as the %RSD values of inter-day precision ranging 0.23–0.47 % for gp120 and 0.21–0.87 % for LL-37. The determined LLOD and LLOQ for gp120 were 0.10 and 0.31 μL mL^−1^, while for LL-37 the values were 0.38 and 1.14 μL mL^−1^, respectively. The assayed accuracy for the lower concentrations approaching the LLOQ of gp120 fragment (at 0.625 μg mL^−1^) and LL-37 (at 1.25 μg mL^−1^) were 114.5 ± 7.5 % and 115.5 ± 0.09 %, respectively (*n* = 3).

### Peptide Extraction from Gels and Peptide Separation by RP-HPLC Method

To evaluate whether current RP-HPLC method was suitable for the quantification of both peptides within polymeric matrices, this method was applied towards the extraction of both gp120 fragment and LL-37 simultaneously from HEC, HPMC, and HPC gels. Results showed that both peptides were successfully extracted from all polymeric gels with high extraction efficiency ranging from 95.90 ± 3.03 to 106.33 ± 2.46 % for gp120 fragment and from 98.27 ± 2.51 to 111.45 ± 2.51 % for LL-37, respectively (Table 2). The loadings of both peptides in the polymeric gel formulations were found in the range 0.0236–0.0241 % for gp120 fragment and 0.0238–0.0248 % for LL-37 using the current extraction procedure (Table 2).

**Table 2:** Extraction of gp120 fragment and LL-37 from different polymeric gels

Type of gels	EE of gp120 (%)	Concentration of gp120 (w/w, %)	EE of LL-37 (%)	Concentration of LL-37 (w/w, %)
1 % HEC gel	95.90 ± 3.03	0.0241 ± 0.0008	98.27 ± 2.51	0.0240 ± 0.0008
1 % HPMC gel	106.33 ± 2.46	0.0236 ± 0.0006	105.66 ± 3.16	0.0238 ± 0.0004
1 % HPC gel	101.07 ± 4.02	0.0241 ± 0.0001	111.45 ± 2.51	0.0248 ± 0.0005

Data represent mean ± SD, *n* = 6

*EE* extraction efficiency (%)

### Identification of gp120 Fragment and LL-37 Degradation/Aggregation Products

Using the current validated method that was developed for quantitating gp120 fragment and LL-37, separation of heat-stressed peptides under acidic exposure was achieved (Fig. 2a, b). There were two distinct gp120 fragment degradants and three LL-37 degradants identified using the current RP-HPLC method (Fig. 2a, b). The induced gp120 fragment degradants were found to have retention times of approximately 2.2 min (G1) and 3.2 min (G2) while LL-37 degradants with retention time of 2.9 min (L1), 4.4 min (L2), and 4.7 min (L3). It was also shown in Fig. 2c that the current method could successfully separate all identified forced degradants from gp120 fragment and LL-37 peaks in the presence of polymers. However, prolonged exposure to stressed conditions (>5 h) resulted in a decrease and/or absence of peaks for gp120 fragment, LL-37, and their forced degraded products (Figs. 2a, c, 3). Further identification of LL-37 forced degraded products revealed that L2 and L3 were approximately 12 and 16 kDa, respectively (Fig. 3a, b).

## Discussion

**Fig. 1:**
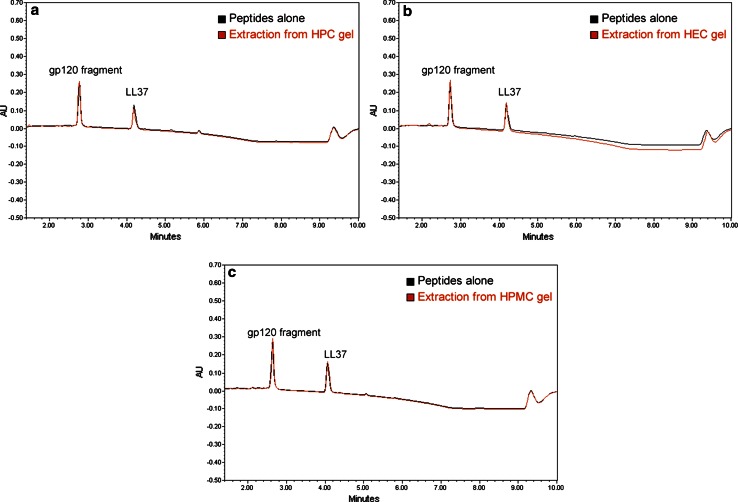
Extraction of gp120 fragment and LL-37 from polymeric gels. Chromatograms of gp120 fragment and LL-37 before and after extraction from **a** HPC, **b** HEC and **c** HPMC gels via the current RP-HPLC method. Each representative chromatogram was obtained from repetitive RP-HPLC analysis (*n* = 6)

In the current study, we have developed an accurate and precise RP-HPLC method with validation for quantitating two peptides simultaneously, gp120 fragment and LL-37, formulated in three polymeric hydrophilic gel formulations with acceptable reproducibility. According to the chromatograms, there was little to no interference caused by the polymeric gels using the current RP-HPLC method with respect to peak shape and peak area (Fig. 1). This could be attributed to the hydrophilic property of the polymers tested in the current study resulting in negligible interactions with the analytical column [11]. In RP-HPLC, hydrophobic molecules (in current study, the two peptides) are first adsorbed onto the hydrophobic stationary phase (e.g., C18 column) in a polar mobile phase and can be later eluted by decreasing the polarity of the mobile phase through the introduction of acetonitrile [11]. Furthermore, our gradient method was capable of simultaneously separating both peptides without any overlap in peaks (Fig. 1). LL-37 and gp120 fragment exhibit different hydrophobicities with an estimated relative hydrophobicity value of 52.62 and 42.16, respectively, as predicated by the current algorithm SSRCalc prediction model (http://hs2.proteome.ca/SSRCalc/SSRCalcX.html, using a 300 Å C18 column in 0.1 % TFA) [33]. This prediction model takes into consideration various parameters which may influence peptide behavior such as amino acid composition, the nature of N- and C-terminal residues, peptide length, and p*I*, etc. [34].Thus, the slightly higher relative hydrophobicity of LL-37 may explain the later elution of this peptide compared to gp120 fragment (Fig. 1).

**Fig. 2:**
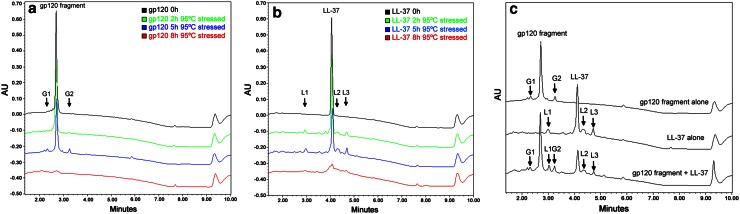
Overlay chromatograms of forced degraded peptides. RP-HPLC overlay chromatograms showing the detection and separation of heat-stressed gp120 fragment alone (**a** heat-stressed up to 8 h), LL-37 alone (**b** heat-stressed up to 8 h), both gp120 fragment and LL-37 analyzed simultaneously comparing to individual peptide chromatograms (**c** heat-stressed for 5 h, spiked into HEC gel) using RP-HPLC. Peaks G1 and G2 represent the force-degraded peaks of gp120 fragment while peaks L1–3 represent LL-37 forced degraded product peaks. The representative chromatogram demonstrated was obtained from repetitive measurements with polymeric gels spiked with both peptides (*n* = 3)

**Fig. 3:**
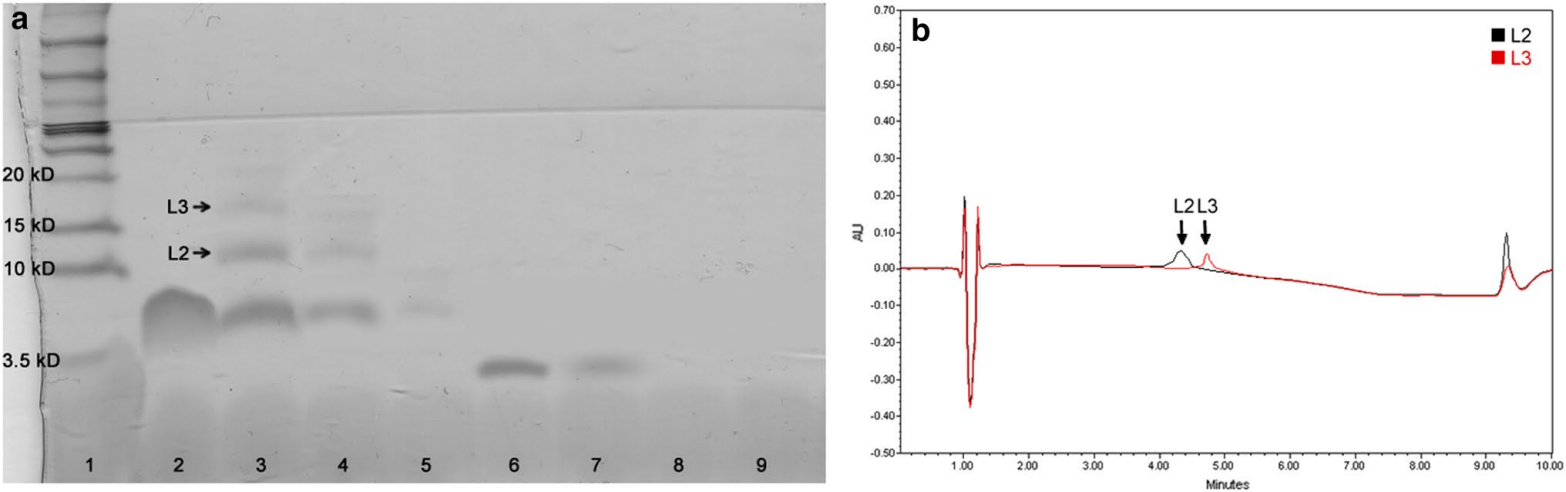
Gel electrophoresis and RP-HPLC detection of forced degraded products. Tris-tricine SDS-PAGE of heat-stressed gp120 fragment and LL-37 samples (**a**) and the overlay chromatogram of LL-37 forced degraded products (**b**).* Lane 1* MW markers;* lane 2* LL-37 non-stressed;* lanes 3–5* LL-37 heat-stressed for 2, 5, 8 h;* lane 6* gp120 fragment non-stressed;* lanes 7–9* gp120 fragment heat-stressed for 2, 5, 8 h. Force-degraded bands (*L2* and* L3*) of LL-37 were extracted from the gel and analyzed using RP-HPLC. Each representative chromatogram was obtained from repeated gel electrophoresis (*n* = 3) and RP-HPLC analysis (*n* = 3)

Since microbicides are usually applied topically to the vagina, peptides formulated in a polymeric pharmaceutical dosage needs to be stable within their surrounding environment, e.g., the physiological temperature of the female genital tract is 37 °C and vaginal fluid is acidic (pH 3.5–4.9) [29]. These conditions may potentially increase the degradation rate of proteins and peptides [29]. Thus, we further evaluated the utility of the current RP-HPLC method to: (1) distinguish forced degradation products of both peptides and (2) quantitate the residual peptides within the polymeric gels without any interference. Under forced degradative conditions for up to 8 h (95 °C in pH 4.2 buffer), the current method was able to differentiate between the degraded products for gp120 (G1–2) and LL-37 (L1–3) (Fig. 2), and was successful in separating the peptide peaks while in the presence of all three polymers (Fig. 2c, a representative spiked HEC gel analysis was presented and similar chromatograms were observed using HPC and HPMC gels). These observations were supported by the tris-tricine SDS-PAGE results (Fig. 3a). Moreover, the current RP-HPLC method was able to identify and differentiate the forced degraded products, which cannot be detected by tris-tricine SDS-PAGE, e.g., products G1, G2, and L1 (Fig. 2). The absent peaks of gp120 fragment, LL-37, and their degradation products after prolonged (>5 h) heat/acid exposure (Fig. 2a, b) could be attributed to the severe degree of hydrolytic cleavage (breaking down into amino acids) of both peptides and the degradants [35, 36].

## Conclusions

In the current study, we developed a fast, accurate, and reliable RP-HPLC method for the quantitation of two candidate microbicide peptides, LL-37 and gp120 fragment simultaneously in polymeric gel formulations. This method was able to detect and separate the forced degraded products of both peptides simultaneously in the presence of polymers without any interference or affecting the determination of peptide concentration, providing a quick and precise alternative to LC–MS techniques.
